# The effect of strain rate on adhesion in the purple sea urchin (*Strongylocentrotus purpuratus*)

**DOI:** 10.1242/bio.062621

**Published:** 2026-08-27

**Authors:** Keegan Lutek, Alyssa Y. Stark

**Affiliations:** ^1^ Department of Biology, Villanova University, 800 Lancaster Ave., Villanova, PA 19085, USA; ^2^ Department of Biology, Acadia University, 33 Westwood Ave., Wolfville, NS B4P 2R6, Canada

**Keywords:** Bodega Marine Reserve, Echinoderm, Intertidal, Marine invertebrate, Material properties, Tube feet

## Abstract

Sea urchins attach to intertidal substrates in the face of dynamic hydrodynamic forces using flexible, adhesive tube feet. Tube-foot-based adhesion varies between species and among populations with different habitats (i.e. temperature and hydrodynamic forces). While some sea urchin species have tube foot stem tissues (tested in anesthetized individuals) that better resist breaking under higher loading rates, it remains unclear whether this improves the whole-animal adhesive performance in unanesthetized animals. To test whether the loading rate and previous hydrodynamic exposure influence the adhesive performance of the intertidal purple sea urchin, *Strongylocentrotus purpuratus*, we measured the strain rate dependence of the tube foot disc, tube foot stem, and whole-animal adhesion, using three sets of sea urchins with different environmental histories. In unanesthetized *S. purpuratus*, strain rate influenced tube foot disc tenacity, but not the tube foot stem or whole-animal adhesion. Rather, sea urchins collected more recently had higher whole-animal adhesion, and reliance on the tube foot disc and the tube foot stem varied among animal sets. These results further support the hypothesis that hydrodynamic loading maintains adhesive performance in sea urchins and highlight the importance of investigating strain rate at levels of organization that impact organismal performance *in situ*.

## Teaser

**Summary:** Adhesive performance is not strain rate dependent (except tube foot disc tenacity) in unanesthetized sea urchins and is higher in purple sea urchins collected more recently from their natural environment.

## INTRODUCTION

In the marine intertidal zone, stochastic wave action varies dramatically with semidiurnal tides, during storms, and across seasons (Denny, 1988; Denny and Gaylord, 1996; Jensen and Denny, 2015). Dynamic, and increasingly extreme, wave action in the intertidal zone generates hydrodynamic lift and drag forces that may dislodge benthic intertidal organisms, potentially causing injury or mortality (Denny, 1988; Jensen and Denny, 2015; Santos and Flammang, 2005; Siddon and Witman, 2003). To overcome these hydrodynamic challenges, many benthic intertidal invertebrates (e.g. sea stars, sea urchins, sea anemones, limpets, and mussels) have evolved unique adhesive strategies to remain firmly attached to the substrate (Babarro and Carrington, 2013; Bell and Gosline, 1996; Bouhlel et al., 2017; Carrington and Gosline, 2004; Carrington et al., 2008; Clarke et al., 2020; Flammang, 1996; Kang et al., 2020; Lengerer and Ladurner, 2018; Moeser et al., 2006; Santos and Flammang, 2005, 2006, 2007, 2008; Santos et al., 2005b; Smith, 1991, 1992; Smith et al., 1993; Thomas and Hermans, 1985; Waite, 2017). These strategies include, but are not limited to, permanent cements and glues (e.g. barnacles and mussels), temporary but strong mucus layers and suction cups (e.g. octopus and limpets), and permanent or temporary mechanically interlocking spines and holdfasts (e.g. kelp and sea urchins).

Sea urchins are benthic marine invertebrates, often found in the intertidal zone, that temporarily move (locomotion) and cling to (adhesion) wet, fouled, and rough substrates in their dynamic environment (Santos et al., 2005b). To accomplish this, they utilize multifunctional hydrostatic structures called tube feet, which are found along the five ambulacra of the sea urchin test (i.e. skeleton; Flammang, 1996). Tube feet on the aboral (top) side of the sea urchin are used for food capture, food manipulation, and respiration, while those on the oral side (bottom) are primarily used for locomotion and adhesion (Lawrence, 1987). In most species, oral tube feet are composed of a stem and a distal adhesive disc. The tube foot stem attaches to the internal water-vascular system, allowing for the tube foot to be extended (via hydrostatic pressure), retracted (via muscular action), and manipulated to accomplish different tasks such as adhering and locomoting with the adhesive disc (Flammang, 1996; Lawrence, 1987). Adhesion of the tube foot disc is generated by two populations of cells on the distal surface, making up a duo-gland adhesive system. Specifically, one set of cells is responsible for secreting a proteinaceous adhesive, while the second set secretes a de-adhesive (likely enzymes) that cleaves the glue at the disc-adhesive interface, allowing the tube foot to be detached from the substrate while moving (Algrain et al., 2022; Flammang, 1996; Flammang et al., 1998, 2016; Gaspar et al., 2021; Lengerer et al., 2019; Santos and Flammang, 2012; Santos et al., 2009; Simão et al., 2020; Ventura et al., 2023; Viana and Santos, 2018).

Whole-animal sea urchin adhesive failure (e.g. via wave-induced loading and predation) can be a result of failure at several scales. First, adhesive tube feet may break, leaving behind adhered adhesive tube foot discs attached to broken tube foot stems (Narvaez et al., 2020, 2022; Santos and Flammang, 2007; Stark et al., 2020). The force needed to break the stem (tube foot stem breaking force, N) is often reported as the breaking force per cross-sectional area (i.e. tube foot stem strength, MPa; Cohen-Rengifo et al., 2017; Garner et al., 2024; Narvaez et al., 2022, 2024; Santos and Flammang, 2005, 2008; Santos et al., 2005b). Tube foot stem strength varies across body locations where the stems of oral tube feet are stronger than the stems of aboral tube feet (Flammang, 1996; Leddy and Johnson, 2000; Narvaez et al., 2022, 2024).

Second, the tube foot discs may detach, leaving behind full or partial adhesive footprints (Narvaez et al., 2020, 2022; Santos and Flammang, 2007). The force required to detach the tube foot disc is measured as the detachment force per unit of adhesive area (i.e. tube foot disc tenacity, MPa; Cohen-Rengifo et al., 2019; Garner et al., 2024; Hennebert et al., 2010; Moura et al., 2023; Narvaez et al., 2022, 2024; Santos and Flammang, 2006, 2008; Santos et al., 2005b). Both tube foot stem strength and tube foot disc tenacity can vary across species and populations (Hennebert et al., 2010; Narvaez et al., 2024; Santos and Flammang, 2005, 2006, 2008; Santos et al., 2005b).

The ability of a sea urchin to adhere to a substrate depends on the material properties of both the tube foot stem and the collective glue/tube foot disc components. Specifically, the primary load-bearing tissue in the tube foot stem is collagen (Santos and Flammang, 2005). Tube foot stem muscles, while present, likely contribute little force, even when active (Santos and Flammang, 2005). Indeed, Santos and Flammang (2025) note that when using values based on the strongest known isometric muscle contraction (the byssus retractor muscle of *Mytilus edulis*), contraction of the muscles of the tube foot stem is estimated to contribute no more than 50% of tube foot stem breaking force. Estimates for relaxed muscles (based on frog striated muscle) suggest they would contribute only 1% of tube foot stem breaking force. Thus, collagen tissue abundance (% cross-sectional area) and collagen molecular state (level of cross-linking), not muscle properties, are the primary influences on the material properties of a tube foot stem. To add further complexity, foot stem strength varies within and among species (Cohen-Rengifo et al., 2017; Leddy and Johnson, 2000; Santos and Flammang, 2005, 2008; Santos et al., 2005b). Further, because tube foot stems contain mutable collagenous tissue (i.e. collagenous tissue under neural control that can shift from ‘soft’ low cross-linking to ‘stiff’ high cross-linking), sea urchins can actively modulate the material properties of the tube foot stem (Santos et al., 2005a). While there are two layers of collagen in the tube foot stem (i.e. outer longitudinal fibers and inner helical fibers), only the longitudinal fibers are understood to be mutable (Santos et al., 2005a).

In contrast to the tube foot stem, tube foot disc adhesion is primarily modulated by material properties (i.e. viscoelastic deformation to facilitate maximum contact area; Santos et al., 2005b) and size (i.e. disc area available for adhesion; Narvaez et al., 2024; Santos and Flammang, 2007, 2008). The material properties and size of the adhesive tube foot disc vary between and within species, leading to differences in tube foot disc tenacity (Cohen-Rengifo et al., 2017; Narvaez et al., 2022, 2024; Santos and Flammang, 2008). Sea urchins may also modulate adhesion via the composition of their adhesive glue (across species; Gaspar et al., 2021) and the total number of adhesive tube feet recruited to resist loading events (i.e. whole-animal behavior; Cohen-Rengifo et al., 2017; Santos and Flammang, 2008). Collectively, the morphological and material properties of tube feet (both the stem and the disc), the composition of the adhesive glue, and the recruitment of tube feet in attachment events (i.e. total number of tube feet deployed to attach) dictate whether a sea urchin can remain attached (i.e. adhesive performance) when experiencing high loads from waves and predation.

Sea urchin adhesive performance also varies in response to the environment. Specifically, species with different habitats have different tube foot stem strength (Santos and Flammang, 2005, 2008), tube foot disc tenacity (Santos and Flammang, 2006, 2008), and maximum adhesive force (of the whole animal, N; Santos and Flammang, 2007, 2008). Likewise, populations from different habitats have different tube foot stem strength (Cohen-Rengifo et al., 2017; Narvaez et al., 2024), tube foot disc tenacity (Cohen-Rengifo et al., 2017; Narvaez et al., 2024), and maximum adhesive force (Cohen-Rengifo et al., 2017; Santos and Flammang, 2007; Stark et al., 2020). In particular, intraspecific and interspecific differences in adhesive performance are related to, and plastic in response to, recent hydrodynamic exposure (e.g. between populations collected from sites with different water velocities, substrates, and likely other parameters or between species found at different depths; Cohen-Rengifo et al., 2017; Laur et al., 1986; Narvaez et al., 2024; Santos and Flammang, 2005, 2006, 2007, 2008; Santos et al., 2005b; Stark et al., 2020).

Hydrodynamic forces in the marine intertidal zone are especially variable. Thus, sea urchin adhesive tube feet are exposed to a wide variety of hydrodynamic force loading rates. These loading rates are commonly expressed as a ‘strain rate’: the rate of deformation of a structure divided by its resting length. For sea urchins, ‘natural’ strain rates (those imposed by the sea urchin itself) are confined to <0.5 s^−1^, while wave-generated strain rates can be significantly higher (≫1 s^−1^) (Denny et al., 1998; Leddy and Johnson, 2000). Previous work has demonstrated that sea urchin tube foot stem tissues (i.e. tube foot stem material properties tested in anesthetized sea urchins) have improved material properties at higher strain rates (Leddy and Johnson, 2000; Santos and Flammang, 2005, 2008). However, strain rate is not always a good predictor of the measured variation in relevant material properties (e.g. tube foot stem breaking force and tube foot stem strength) as its influence varies between species.

Positive strain rate dependence of tube foot stem breaking force, tube foot stem strength, tube foot stem toughness (i.e. the energy required to break a tube foot stem, MJ/m^3^), and other material properties could increase the dissipation of wave energy on the whole animal. However, interspecies differences in the strength of the relationship between strain rate and these material properties call into question the overall importance of strain rate on sea urchin adhesion. For example, in *Paracentrotus lividus*, higher strain rates increase tube foot stem strength, but only 4% of the measured variation in tube foot stem strength was explained by changes in strain rate (Santos and Flammang, 2006). In contrast, in *Strongylocentrotus droebachiensis*, about half of the variation in tube foot stem breaking force, tube foot stem breaking strength, tube foot stem extensibility (i.e. length at breaking divided by resting length), and tube foot stem breaking stiffness (i.e. slope of a stress-strain curve, MPa) was explained by strain rate (Leddy and Johnson, 2000). Finally, in four sympatric tropical species (*Colobocentrotus atratus*, *Echinometra mathaei*, *Heterocentrotus trigonarius*, and *Stomopneustes variolaris*), tube foot stem extensibility (>41% measured variation explained by strain rate), tube foot stem toughness (17-28% measured variation explained by strain rate), and (for one species) tube foot stem strength were positively related to strain rate (Santos and Flammang, 2008). Further, all of these experiments were done at relatively low strain rates (<1 s^−1^) on anesthetized sea urchins, thereby measuring tissue material properties without muscle contraction or stiffened mutable collagenous tissue. Thus, it is unclear whether strain rate dependence is common across sea urchin species, whether such dependence extends to higher rates of strain (which would be commonly imposed by waves), or if strain rate dependence is meaningful in unanesthetized animals.

**Fig. 1.**
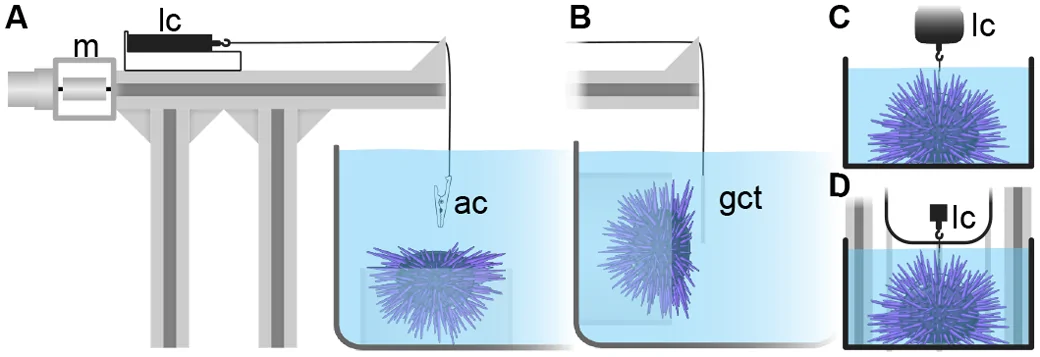
**Experimental apparatus for testing the properties of tube foot stems, tube foot discs, and whole-animal adhesion.** (A,B) Tube foot stem properties (A) and tube foot disc properties (B) were measured using the same apparatus, but with a different attachment at the end. Briefly, once a tube foot stem was captured in an alligator clip (ac; A) or a tube foot disc attached to a glass capillary tube (gct; B), the load cell (lc) was pulled toward the motor (m) such that attachment moved vertically away from the urchin. (C,D) Whole-animal adhesion was measured by pulling an urchin vertically off a glass surface either by hand (C) or via a custom force apparatus (D). Example videos of these tests can be found in Movie 1.

To test the effect of strain rate, at multiple scales, on unanesthetized sea urchin adhesive performance, we used a common intertidal sea urchin, the purple sea urchin (*Strongylocentrotus purpuratus*), that regularly experiences high wave forces. Specifically, we measured the strain rate dependence of tube foot stem material properties (tube foot stem breaking force; tube foot stem extension, the length of the tube foot stem at breaking, m; tube foot stem spring constant, the slope of a force-extension curve, N m^−1^; and tube foot stem work to failure, the area under a force-extension curve, J), tube foot disc tenacity, and adhesive performance of the whole animal (maximum adhesive force and whole-animal adhesion, maximum adhesive force divided by attachment area, MPa) in three sets of sea urchins with different environmental histories (CA8: California sea urchins tested 8 weeks after collection; CA44: California sea urchins tested 44 weeks after collection; and WA2: Washington sea urchins tested 2 weeks after collection) (Fig. 1). The tested strain rates span both natural extension and wave-induced strain rates, and our measurements were completed using unanesthetized sea urchins. We predict that tube foot disc tenacity, tube foot stem material properties, and therefore adhesive performance of the whole animal (i.e. maximum adhesive force and whole-animal adhesion) will have a positive relationship with strain rate, providing additional resistance to breaking and potentially devastating detachment in the wave-swept intertidal zone. Further, we predict that all measured variables will be higher for sea urchins with more recent exposure to natural wave action. The results of this study will improve our understanding of the impact that loading rate has on adhesive performance in an ecologically important marine invertebrate.

## RESULTS

**Fig. 2.**
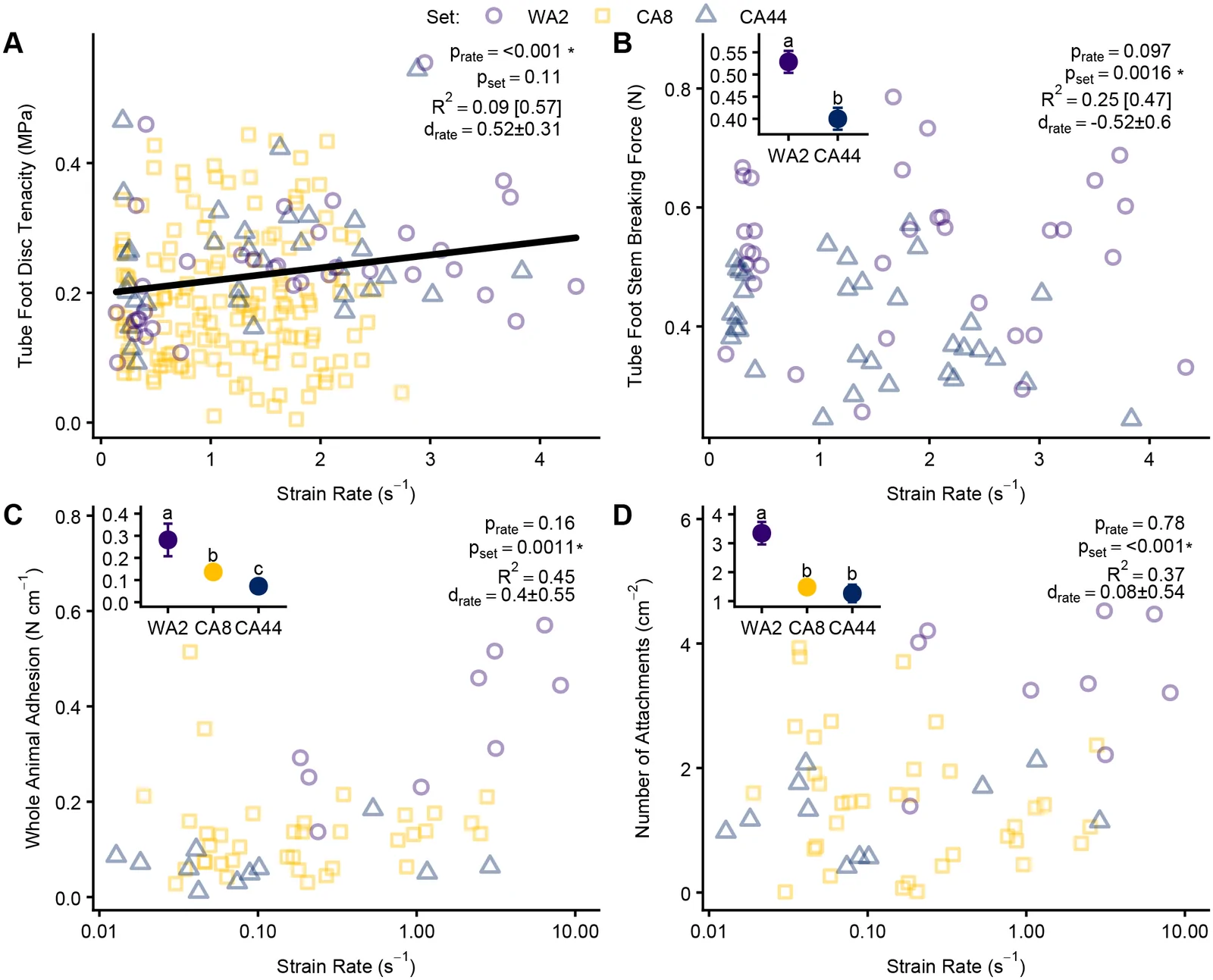
**Strain rate has limited influence on tube foot and whole-animal adhesive performance.** (A) Tube foot tenacity (slope for strain rate±s.e.m.=0.020±0.006 MPa s) was measured as the maximum adhesive force of a single tube foot divided by the average tube foot disc area for a sea urchin (*n*=44). (B) Tube foot stem breaking force was measured as the maximum force required to break a tube foot stem (*n*=22). (C) Whole-animal adhesive force was measured as the maximum force recorded while pulling a sea urchin from a glass substrate divided by the maximum area available for attachment (*n*=61). (D) The number of attachments was counted (both footprints and broken tube feet) after sea urchins were pulled from a glass substrate and divided by the maximum area available for attachment (*n*=61). When significant differences between animal sets are present for non-interaction models (B-D), insets show these differences (estimated marginal mean±s.e.m.) at the mean strain rate (different letters denote statistically significant differences; post-hoc Tukey tests, *P*<0.05). Note that the *x*-axes in C and D are log transformed to better show observations at low strain rates. Points are individual observations. Lines of best fit are shown only when strain rate was a significant predictor (A). Only the pooled estimate of the effect of strain rate is plotted in panel A because there is no statistically significant effect of animal set on tube foot tenacity. The two *R*^2^ estimates in A and B represent the variance explained by fixed effects (marginal *R*^2^; before square brackets) and variance explained by the whole model (i.e. fixed and random effects; conditional *R*^2^; inside square brackets). Asterisks indicate statistically significant *P*-values (*P*<0.05). Letters in the set names refer to collection location (CA: California, WA: Washington), and numbers in the set names refer to the time spent out of their natural habitat prior to experiments, in weeks. Full description of animal sets is available in the text. Complete statistical output is available in Table 2. d_rate_: effect size for the effect of strain rate on each dependent variable, along with the 95% confidence interval.

Descriptive statistics for the adhesive performance of the three sets of sea urchins (CA8, CA44, and WA2) are shown in Table 1. There was a statistically significant positive effect of strain rate on tube foot disc tenacity (slope=0.020±0.006 MPa s; F_1,171_=11.39; *P*<0.001), and there were no differences in this relationship between any of the animal sets (F_2,42_=2.34; *P*=0.11; Fig. 2A, Table 2). However, the fixed effects of strain rate and animal set explain only 9.2% of the variation in tube foot disc tenacity, and the effect size for strain rate (d_strain_) was moderate, with a wide confidence interval (0.52±0.31; Fig. 2A, Table 2). Strain rate did not have a statistically significant effect on tube foot stem breaking force (slope=−0.018±0.011 N s; F_1,43_=2.89; *P*=0.097; d_strain_±CI=−0.52±0.6), whole-animal adhesion (slope=0.102±0.071; F_1,52_=1.98; *P*=0.16; d_strain_±CI=0.4±0.55), or number of attachments (slope=0.029±0.105; F_1,52_=0.08; *P*=0.78; d_strain_±CI=0.08±0.54; Fig. 2B-D, Table 2). Tube foot stem breaking force (F_1,20_=13.35; *P*=0.0016) was higher for the WA2 set than for the CA44 set (Fig. 2B, Table 2). Whole-animal adhesion and number of attachments also varied across animal sets (F_2,52_=7.84; *P*=0.0011 and F_2,52_=9.90; *P*<0.001, respectively; Fig. 2C,D, Table 2). Sea urchins in the WA2 and CA8 sets adhered more strongly than those in the CA44 set (post-hoc Tukey comparisons; *P*=0.0007 and *P*=0.022, respectively; Fig. 2C), and the WA2 set adhered more strongly than the CA8 set (post-hoc Tukey comparison; *P*=0.049; Fig. 2C). The number of attachments made to the substrate was higher for the WA2 set than for both the CA8 and the CA44 sets (post-hoc Tukey comparisons; *P*=0.0003 and *P*=0.0004, respectively; Fig. 2D), but there was no significant difference between the CA8 and CA44 sets (post-hoc Tukey comparison; *P*=0.797; Fig. 2D).

**Table 1.** Descriptive statistics for adhesive performance of purple sea urchins on glass

Variable	WA2	CA8	CA44
Tube foot disc tenacity (MPa)	0.240 [0.092, 0.555]	0.191 [0.005, 0.444]	0.253 [0.092, 0.544]
Tube foot stem breaking force (N)	0.52 [0.26, 0.79]	−	0.40 [0.24, 0.57]
Tube foot stem extension (mm)	4.5 [2.7, 7.9]	−	3.6 [1.3, 7.0]
Tube foot stem initial spring constant (N m^−1^)	1.01 [0.01, 2.96]	−	1.39 [0.41, 3.03]
Tube foot stem final spring constant (N m^−1^)	30.5 [1.1, 40.6]	−	37.5 [16.3, 81.7]
Tube foot stem work to failure (J)	0.018 [0.006, 0.047]	−	0.011 [0.002, 0.023]
Number of attachments (cm^−1^)	3.40 [1.39, 4.52]	1.38 [0.01, 3.93]	1.25 [0.41, 2.12]
Maximum adhesive force (N)	31.0 [11.4, 48.3]	11.8 [2.6, 45.4]	6.3 [1.1, 19.2]
Whole-animal adhesion (N cm^−2^)	0.33 [0.13, 0.57]	0.13 [0.03, 0.51]	0.07 [0.01, 0.18]

Values presented for each set are the mean and range (square brackets). Note that these values ignore strain rate.

**Table 2.** Model parameters for adhesive performance of purple sea urchins on glass

Response	Form	Type	*n*	Term	*F*	d.f.	*P*	*R* ^2^
Tube foot disc tenacity (MPa)	rate+set	Lme	45	rate	11.39	1, 171	**<0.001**	0.09 [0.57]
				set	2.34	2, 42	0.11	
Tube foot stem breaking force (N)	rate+set	Lme	22	rate	2.89	1, 43	0.097	0.25 [0.47]
				set	13.35	1, 20	**0.0016**	
Number of attachments (cm^−2^)	rate+set	Lm	61	rate	0.08	1, 52	0.78	0.37
				set	9.90	2, 52	**<0.001**	
Whole-animal adhesion (N cm^−2^)	rate+set	Glm	61	rate	1.98	1, 52	0.16	0.45
				set	7.84	2, 52	**0.0011**	
	fp×set	Glm	61	fp	3.78	1, 54	0.057	0.68
				set	14.68	2, 54	**<0.0001**	
				fp:set	13.03	2, 54	**<0.0001**	
	btf×set	Glm	61	btf	8.64	1, 54	**0.0048**	0.52
				set	7.08	2, 54	**0.0019**	
				btf:set	3.96	2, 54	**0.025**	

For linear mixed-effects models, both the marginal (fixed effects only) and conditional (full model; in square brackets) *R*^2^ values are presented. Statistically significant *P*-values are in bold. The effect sizes for strain rate in each of these models (unless there is an interaction, which precludes a meaningful estimate for strain rate alone) are presented in Fig. 2. btf, broken tube feet; d.f., degrees of freedom; fp, footprints; Lm, linear model; Lme, linear mixed effects model; Glm, generalized linear model; *n*, number of individuals in the model; rate, strain rate; set, animal set (i.e. CA8, CA44, or WA2; see text for the full description of animal sets).

**Fig. 3.**
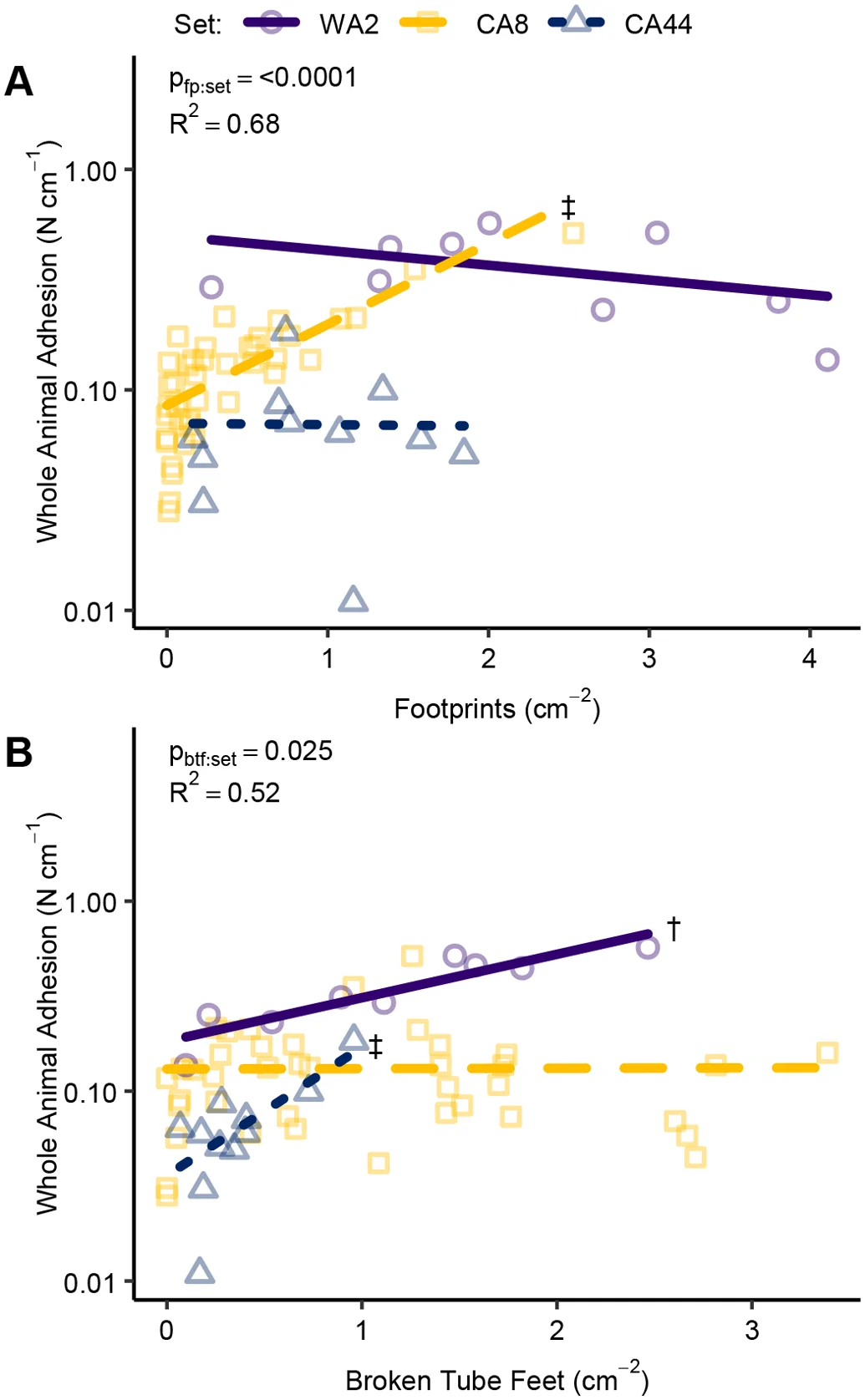
**The influence of footprints and broken tube feet on whole-animal adhesion depends on the group of animals tested.** Whole-animal adhesive performance was measured as the maximum adhesive force when pulled from a glass substrate divided by the theoretical maximum area available for attachment (*n*=61). (A,B) Footprints (A) and number of broken tube feet (B) were counted following each pull and normalized to the theoretical maximum area available for attachment. Points are individual observations. Lines of best fit are as determined by generalized linear models. ^†^*P*<0.1 and ^‡^*P*<0.05 indicate lines with a nonzero slope (simple slope test). Letters in the set names refer to collection location (CA: California, WA: Washington), and numbers in the set names refer to the time spent out of their natural habitat prior to experiments in weeks. Full description of animal sets is available in the text. Complete statistical output is available in Table 2.

Whole-animal adhesion was explained by the interaction between animal set and number of footprints (F_2,54_=13.03; *P*<0.0001) and by the interaction between animal set and the number of broken tube feet (F_2,54_=3.96; *P*=0.025; Fig. 3, Table 2). Specifically, whole-animal adhesive performance in the CA8 set is well predicted by the number of footprints (simple slope test; *P*_WA2_=0.23, *P*_CA8_≤0.0001, *P*_C44_=0.95), whereas whole-animal adhesive performance for the WA2 and the CA44 sets is better predicted by the number of broken tube feet (simple slope test; *P*_WA2_=0.06, *P*_CA8_=0.97, *P*_C44_=0.04; Fig. 3).

None of the tube foot tensile parameters (tube foot stem breaking force, tube foot stem extension, tube foot stem initial spring constant, tube foot stem final spring constant, and tube foot stem work to failure) were significantly affected by strain rate (Fig. S1, Table S1). Tube foot stem extension and tube foot stem work to failure were significantly higher in the WA2 set than in the CA44 set (Fig. S1, Table S1).

## DISCUSSION

Most biological materials are viscoelastic; their ability to resist loading depends on the rate at which the load is applied. Previous work has demonstrated that, in anesthetized animals, the tube foot disc is viscoelastic, and the tube foot stem resists higher loading rates more strongly than lower loading rates (Leddy and Johnson, 2000; Santos and Flammang, 2005, 2008; Santos et al., 2005b). However, in live, unanesthetized animals, it is unclear if this strain rate dependence is maintained since there may be changes in attachment behavior and changes in mutable collagenous tissue state. The results of this study using unanesthetized sea urchins show that while tube foot disc tenacity is strain rate dependent, the material properties of the tube foot stem and whole-animal adhesion are not. We do, however, find notable differences in the adhesive performance and behavior of sea urchins with different environmental histories.

The results of this study found a small, but statistically significant, effect of strain rate on tube foot disc tenacity (Fig. 2A). Tube foot disc tenacity (maximum adhesive force of the tube foot disc per unit area) as presented here (and elsewhere; Cohen-Rengifo et al., 2017, 2018; Narvaez et al., 2020; Santos and Flammang, 2006, 2007, 2008; Santos et al., 2005b; Stark et al., 2020) reflects (1) the material properties of the tube foot disc, (2) the properties of the tube foot disc adhesive glue, and (3) the propensity of the sea urchin to voluntarily detach from a substrate. Although the material properties of the tube foot disc are not well studied, they appear to be viscoelastic like the tube foot stem (Santos et al., 2005b). The molecular composition of echinoderm adhesives is an area of active investigation, where current evidence suggests that polysaccharides are an important component of the sea urchin disc adhesive (Viana and Santos, 2018; Santos et al., 2009; Flammang and Jangoux, 1993; Gaspar et al., 2021) and that polysaccharide matrices are viscoelastic (Smith, 2002). Thus, it is not surprising that our results demonstrate a statistically significant relationship between tube foot disc tenacity and strain rate. As in previous investigations (Cohen-Rengifo et al., 2017; Cohen-Rengifo et al., 2018; Narvaez et al., 2020; Santos and Flammang, 2006, 2007, 2008; Santos et al., 2005b; Stark et al., 2020), we cannot separate the influence of the adhesive and of the possibility that the unanesthetized sea urchin has voluntarily detached from the substrate. However, regardless of the mechanism (i.e. glue composition or behavioral choice), the testing methods used here reflect the best estimate of maximal adhesive performance of an individual tube foot disc and suggest that tube foot disc tenacity varies with strain rate in the purple sea urchin.

Perhaps more perplexing is that the assumed strain rate dependence of the tube foot stem tissues does not manifest in unanesthetized purple sea urchin tube foot stems (tube foot stem breaking force, tube foot stem extension, tube foot stem initial spring constant, tube foot stem final spring constant, and tube foot stem work to failure; Fig. 2B; Fig. S1). Although the stems of sea urchin tube feet generally appear to share morphological and tissue similarities (at least in terms of the relative percentages of tissue layers; Santos and Flammang, 2005, 2008), it may be that the amount of or structure of the collagenous tissue in purple sea urchins is different from the species investigated to date. Even among those species with similar tissue percentages, tube foot stem material properties can vary (Santos and Flammang, 2005, 2008), possibly as a result of the chemical and structural organization of the collagenous tissue. It would therefore be informative to investigate the tissue composition and stem morphology of purple sea urchin tube feet and compare this to other species and their associated material properties.

Perhaps equally, if not more, important may be the state of the unique mutable collagenous tissue when a sea urchin is anesthetized or unanesthetized. When the animal is unanesthetized, mutable collagenous tissue in the tube foot stem is expected to be in the ‘stiff’ configuration, and muscles are expected to be active (Santos and Flammang, 2005). While mutable collagenous tissue is significantly stronger in the ‘stiff’ configuration than in the ‘soft’ configuration (Santos and Flammang, 2005), the only difference between these states is the level of cross-linking between collagen fibers. However, polymers that are more heavily cross-linked may be less strain rate sensitive because this cross-linking limits the movement of the polymer fibers against each other. If this is also true of mutable collagenous tissue, it would explain the disparity between our results in unanesthetized sea urchins and those of previous work, which tested the stem tissue in a ‘soft’ state (i.e. anesthetized). Further, our results draw attention to the roles of the longitudinal and helical collagen fibers when external load is applied to the tube foot stem. Especially under extreme loads, the helical fibers are likely to contribute longitudinal resistance to the tube foot stem (which they would not do in their slack state). Since these fibers are not mutable, it is likely that they would remain strain rate dependent (if our supposition above holds) and therefore impart strain-rate-dependent properties to the tube foot stems of sea urchins. Given that we do not see any strain rate dependence in the tube foot stem material properties, the helical fibers may be contributing little to no overall longitudinal resistance, or they themselves are not strain rate dependent.

Previous work estimates that the contribution of muscle to tube foot stem breaking force is of negligible (i.e. no more than 50% of total resistance to breaking) importance for sea urchins (Santos and Flammang, 2005). The classic force-velocity curve of muscles predicts that muscle force during eccentric contractions is positively related to strain rate (Edman, 1988). Since our results found no strain rate dependence of the tube foot stem material properties, even when muscles would be active during eccentric contraction and thus could contribute more force due to the force-velocity curve, our result is in accordance with the prediction that muscles do not contribute meaningfully to tube foot stem breaking force.

Despite the influence of strain rate on tube foot disc tenacity, we found no effect of strain rate at the whole-animal level (i.e. whole-animal adhesion or number of attachments; Fig. 2C,D). These results hold even for rates of strain that begin to approach those imposed by wave action (and across a larger range of strain rates than previous work has tested; Leddy and Johnson, 2000; Santos and Flammang, 2005, 2008). *S.* *purpuratus* inhabits wave-swept and highly hydrodynamic environments (Hodin et al., 2020). These environments likely select for high adhesive performance. Indeed, the whole-animal maximum adhesive forces we recorded in the WA2 set (Table 1) are among the highest in the literature for whole-animal maximum adhesive force (the only other comparable values reported were from *Sphaerechinus granularis*; Santos and Flammang, 2007). It may be that selective pressures on *S. purpuratus*, perhaps in part because of their high-force environment, have favored absolute adhesive performance at the expense of strain rate dependence.

Purple sea urchins are plastic in response to a variety of abiotic conditions, and, notably, their adhesive performance at the level of the tube foot disc, the tube foot stem, and the whole animal is plastic in response to hydrodynamic conditions (Cohen-Rengifo et al., 2017; Narvaez et al., 2022; Santos and Flammang, 2007). Adhesive performance between sea urchin species also correlates well with habitat, suggesting that species accommodate their abiotic and hydrodynamic environment through a combination of evolution and plasticity. Further, previous work has demonstrated that quiescent conditions and time in the laboratory reduce adhesive performance and change adhesive morphology across sea urchin species (Cohen-Rengifo et al., 2019; Narvaez et al., 2024; Santos and Flammang, 2007). Thus, it seems that adhesive performance requires hydrodynamic loading to be maintained. The sea urchins that we used came from two populations (WA2 from a single location in Washington; CA8 and CA44 from a single location in California), had three acclimatization histories, and spent different durations out of their natural environment. Our results are consistent with the hypotheses that adhesive performance requires hydrodynamic forces to be maintained and that holding sea urchins in the lab reduces their adhesive performance because sets with more recent exposure to environmental hydrodynamics have higher whole-animal adhesion (WA2>CA8>CA44). However, given that hydrodynamics is not the only factor that differs between our animal sets (e.g. water chemistry, tidal cycle, and genetics), these differences may be due to other factors as well. Looking at intraspecific differences in adhesive performance at both constant and varying strain rates between habitats with different hydrodynamic conditions would yield critical insights into the adhesive biology and performance of sea urchins.

Animal sets also differed in relation to number of attachments (footprints or broken tube feet) left behind after a whole-animal adhesion test. These results suggest that sea urchins may adjust to the imposed force by changing the number of tube feet that they use to adhere to a substrate. Notably, however, whether footprints or broken tube feet best predicted whole-animal adhesion did not vary in a predictable way with environmental history (i.e. animal set). Specifically, whole-animal adhesion in the CA8 set was well predicted by the number of footprints, while whole-animal adhesion in the CA44 set and the WA2 set was well predicted by the number of broken tube feet. It is therefore unclear why different animal sets use different strategies to achieve their adhesive performance. Sea urchins are known to exhibit a variety of behavioral responses to increased hydrodynamic force (Cohen-Rengifo et al., 2018; George and Carrington, 2014; Stewart and Britton-Simmons, 2011). Our work supports the idea that sea urchins can modulate the number of attached tube feet based on the perceived force (Cohen-Rengifo et al., 2018) since the number of attachments varied between animal sets. However, this may be an acclimation (or acclimatization) effect that requires some period of time, rather than being an immediate response, as we found no relationship between the number of attachments and the strain rate.

Our work investigates the influence of strain rate on adhesive performance in unanesthetized purple sea urchins across a range of strain rates that extend beyond ‘natural’ extension rates. We found strain rate dependence at the tube foot disc (i.e. tube foot tenacity), but not at the tube foot stem (i.e. tube foot stem breaking force, tube foot stem extension, tube foot stem initial and final spring constants, and tube foot stem work to failure), in whole-animal adhesion, or in the number of attachments. Instead, we found that adhesive performance is higher in experimental sets with more recent exposure to environmental hydrodynamics, and we found that the role of the tube foot disc and tube foot stem differs among animal sets. These results highlight the influence of environmental history on adhesive performance, the importance of investigating strain rate dependence at all levels of the tube foot system in active animals, and the potential role for behavior to determine sea urchin adhesive performance.

## MATERIALS AND METHODS

### Animal collection and husbandry

Adult *S.* *purpuratus* were collected and tested between November 2022 and July 2024. Sea urchins from Pt. Arena Cove (Arena Cove, CA, USA) were collected by the Aquatic Resources Group at Bodega Bay Marine Laboratory in November 2022. Immediately following collection, sea urchins were moved to a flow-through artificial tidepool (Bodega Marine Laboratory, Bodega Bay, CA, USA) fed by a single-pass seawater system and held there until experiments began (approximately 8 weeks). We performed two sets of experiments using sea urchins from this California collection (CA): one at Bodega Bay in January 2023 (8 weeks after removal from their natural environment) and a second at Villanova University in October 2023 (44 weeks after removal from their natural environment). For clarity, we will hereafter refer to these two animal sets by their collection location and time out of their natural environment: ‘CA8’ and ‘CA44’, respectively. The third set of adult sea urchins was collected from the intertidal zone at Clallam Bay (WA, USA) in June 2024. Sea urchins were held overnight in coolers and then transported to Friday Harbor Laboratories, where they were held in a single-pass seawater system until experiments began (approximately 2 weeks). Like the sets from CA, we will hereafter refer to these animals as ‘WA2’, which jointly identifies their collection location and time out of their natural environment.

Sea urchins in set CA8 (*n*=37, mass=117.31±21.58 g, diameter=63.73±0.80 mm, height=34.54±0.57 mm) were moved from the artificial tidepool into hanging mesh bags (to avoid tube foot damage) in a single-pass seawater table for the duration of experiments (less than 2 days). Sea urchins were not fed while in the flow-through seawater table.

Sea urchins in set CA44 (*n*=11, mass=94.55±8.38 g, diameter=63.233±1.71 mm, height=33.75±1.59 mm) were shipped overnight from the Bodega Marine Laboratory to Villanova University. At Villanova University, animals were housed in artificial seawater (31 ppt, 10°C) in hanging bags in 10-20 gallon tanks. Water changes were performed daily to ensure water quality, and experiments were performed within 4 days of their arrival. Sea urchins were not fed during that time.

Sea urchins in set WA2 (*n*=12, mass=117.25±5.56 g, diameter=66.16±1.11 mm, height=35.77±0.91 mm) were kept in hanging mesh bags (single-pass seawater system; 31 ppt, 11.5°C) and fed *ad libitum*.

### Tube foot morphology

For all animal sets, tube foot stem length and tube foot disc area were assessed in the image processing software Fiji (Schindelin et al., 2012) using the protocol of Narvaez et al. (2020). Specifically, to measure the tube foot stem length, individuals were immobilized in a sponge and placed into a 13 cm-diameter PVC pipe affixed to the side of a 6-l seawater-filled (CA8, WA2) or artificial-seawater-filled (CA44) plastic container. The oral tube feet were allowed to extend, and the sea urchin was imaged (Nikon Z30 with Nikon Nikkor DX 16-50) with a ruler placed through the field of view. Five to ten extended and in-focus tube feet were measured (i.e. the total length from the base of the tube foot to the distal disc) to calculate an average tube foot length for each individual. To measure tube foot area (i.e. the adhesive epidermis and the peripheral disc of each tube foot), sea urchins were immobilized in a sponge, inverted (i.e. oral side up) and submerged in a 6-l plastic container. A glass Petri dish lid with a scale bar was floated on the surface of the water over the sea urchin. Once at least ten tube feet had visibly attached to the glass Petri dish, the sea urchin was photographed, and the area of at least ten attached tube foot discs was measured and used to calculate the average tube foot disc area of each individual.

### Tube foot stem material properties and tube foot disc adhesive performance

Individual tube foot stem material properties were measured for the CA44 (*n*=11) and WA2 (*n*=12) sets using a custom-built apparatus (Fig. 1A; Garner et al., 2024). This apparatus consists of a small metal alligator clip with sandpaper affixed to each inner side, used to hold the tube foot stem, attached to a 100 g JR S-Beam Load Cell (LSB200, FUTEK, Irvine, CA, USA) force transducer on a horizontal moveable sled that was moved by an Arduino Uno (Arduino, Somerville, MA, USA) controlled motor, such that the attachment substrate (the alligator clip) could be moved at controlled rates (Fig. 1A). Specifically, sea urchins were immobilized oral side up in a sponge in a PVC pipe affixed to the bottom of a plastic container (i.e. the same configuration used for tube foot disc area measurements). Tube feet were allowed time to extend, and an individual tube foot was captured in the metal clip. Approximately 1-2 mm of the stem was secured in the clip, enough that the adhesive tube foot disc was positioned in the center of the clip so that it was held firmly by the sandpaper and clip. The clip was then pulled vertically away from the sea urchin at a constant rate. Each individual was tested three times (different tube feet) at the following loading rates: 0.45, 2.35 and 4.15 cm s^−1^. We used only the maximum force to break a tube foot stem, identified using custom MATLAB (MathWorks, Portola Valley, CA, USA) code from traces recorded using SENSIT 2.7 (FUTEK), for data analysis (i.e. not the average; Narvaez et al., 2022, 2024). Since experimental logistics precluded measurement of tube foot stem connective tissue cross-sectional area, we report ‘tube foot tensile behavior’ (as in Garner et al., 2024) in place of standard measures to describe the material properties of the tube foot stems. We therefore report tube foot stem breaking force (the maximum force recorded during a stem pull, N; analogous to tube foot stem strength), tube foot stem extension (the change in length between when force reached 0.0015 N and when it reached breaking, m; Santos and Flammang, 2005; analogous to extensibility), tube foot stem initial spring constant (the slope of the initial linear region of a force-extension plot, N m^−1^; analogous to stiffness), tube foot stem final spring constant (the slope of the final linear region of a force-extension plot, N m^−1^; analogous to stiffness), and tube foot stem work to failure (the area under a force-extension plot between when force reached 0.0015 N and when it reached tube foot stem failure, J; analogous to toughness).

In addition to tube foot stem material properties, we also measured individual tube foot disc adhesive performance (CA8: *n*=21, CA44: *n*=11, WA2: *n*=12) using the same custom-built apparatus detailed above, but with a glass capillary tube in place of the alligator clip (Fig. 1B; Garner et al., 2024). To initiate attachment, sea urchins were restrained in a sponge, and a PVC pipe was attached to the side of a plastic container, as described above, and allowed time to extend their tube feet. The glass capillary tube was then placed in front of the extended oral tube feet. Once a single tube foot had attached to the capillary tube and the tube foot stem had started to retract, the capillary tube was pulled vertically away from the attached tube foot at a constant rate. We recorded the force trace and identified the maximum tube foot adhesive force with the same analysis procedure used for tube foot stem breaking force. For CA8, three trials per individual (using different tube feet) were performed at each of the following loading rates: 0.45, 1.1, 1.7, 2.35, 3.1, 3.6, and 4.15 cm s^−1^. For the CA44 and WA2 sets, we similarly performed three trials per individual using different tube feet, but only at three rates (i.e. 0.45, 2.35, and 4.15 cm s^−1^) to minimize stress on the sea urchins as we were performing additional tube foot stem measurements in these experiments. Only the maximum recorded adhesive force of the three trials was used for statistical analysis (Moura et al., 2023; Narvaez et al., 2020, 2022, 2024). Tube foot disc tenacity (MPa) was calculated by dividing the recorded maximum tube foot adhesive force at each strain rate by the average tube foot disc area for that individual.

Strain rate was calculated by dividing loading rate by the average length of each individual's tube feet (strain rate, s^−1^). Strain rate order was randomized for each individual to minimize any effects of testing order, and all individuals were tested at all rates for each set.

### Whole-animal force measurements

To measure whole-animal adhesive performance, sea urchins (CA8: *n*=35, CA44: *n*=11, WA2: *n*=9) were affixed with a four-point harness (to distribute force and avoid peeling the sea urchin off the glass) and allowed to adhere to a 20×20 cm glass square submerged in natural seawater (CA8 and WA2) or artificial seawater (CA44) for 1 min prior to pulling the sea urchin vertically from the glass (Fig. 1C,D; Stark et al., 2020). During this 1-min period, the sea urchin was agitated and gently tensioned against the substrate to induce adhesion. For the CA8 and the CA44 sets, the sea urchins were then pulled by hand (Fig. 1C) at a range of rates (CA8: 0.04-5.2 cm s^−1^; CA44: 0.03-5.1 cm s^−1^), and maximum adhesive force (N) was measured by a 200 N FGV-50XY force gauge (Nidec, Kyoto, Japan). For the WA2 set, sea urchins were pulled at one of three loading rates (0.27, 3.46, or 9.57 cm s^−1^) using a custom-built force apparatus (Fig. 1D), and the maximum adhesive force was recorded using a 44.82 N Submersible Miniature S-Beam Jr. Load Cell (LSB210, FUTEK). We then calculated whole-animal adhesion as the measured maximum adhesive force divided by the surface area available for adhesion (i.e. the oral half of the total surface area of the sea urchin test, estimated as an oblate spheroid, N cm^−2^; Stark et al., 2020). All hand-pulled trials were video recorded at 30 fps (Tough TG-5, Olympus, Shinjuku City, Tokyo, Japan) to allow post-hoc determination of loading rate and strain rate. Loading rate was measured by digitizing the position of the force transducer in DLTdv8 (Hedrick, 2008). Strain rate was calculated by dividing the loading rate by the average length of each individual's tube feet.

Finally, to evaluate the role that adhesive failure (footprints) and tube foot stem failure (broken tube feet) play in whole-animal adhesive force, we counted the number of footprints and broken tube feet following each whole-animal adhesive performance test. Directly following whole-animal adhesion testing, the square of glass used as the adhesive substrate was transferred to Crystal Violet (0.05% in tap water) for 4 min to stain sea urchin tube foot disc footprints and broken tube feet (Santos and Flammang, 2006; Stark et al., 2020). The number of footprints (cm^−2^) and the number of broken tube feet (cm^−2^) were counted by hand from the Crystal Violet stain images using the multipoint tool in Fiji and divided by the surface area available for adhesion (Stark et al., 2020). We also calculated the number of attachments (cm^−2^) as the sum of the number of footprints and the number of broken tube feet. Finally, at the completion of experiments, the sea urchin height, diameter, and wet weight were measured, and the sea urchin was returned to its housing.

### Statistical analysis

To assess the relationships between strain rate and (1) tube foot stem breaking force, (2) tube foot stem extension, (3) tube foot stem initial spring constant, (4) tube foot stem final spring constant, (5) tube foot stem work to failure, and (6) tube foot disc tenacity, we used linear mixed effects models generated in the R package nlme (https://CRAN.R-project.org/package=nlme). Strain rate and animal set were included as fixed effects, and the individual was modeled as a random intercept. *R*^2^ values (both marginal, fixed effects only, and conditional, full model) for these linear mixed effects models were estimated using the MuMIn package (https://CRAN.R-project.org/package=MuMIn). The relationship between the number of attachments and strain rate was assessed with a linear regression. The relationships between whole-animal adhesion and strain rate, whole-animal adhesion and number of footprints, and whole-animal adhesion and number of broken tube feet were assessed with generalized linear models to account for non-normal residuals. For all variables, an interaction term was included only when the Akaike information criterion (AIC) value for the interaction model was at least two units lower than the AIC value for the non-interaction model. The assumptions of all models were checked visually. We report Cohen's *d* (Cohen, 2013), as well as estimates of the 95% confidence interval about that effect size, for the effect of strain rate in any non-interaction models that include strain rate to quantify the magnitude of strain rate dependence in each measurement. Values of Cohen's *d* that are ≤0.2 are considered ‘small’ (i.e. not biologically meaningful), and those ≥0.8 are considered ‘large’ (i.e. biologically meaningful; Cohen, 1988). The 95% confidence intervals provide information about the precision of these effect size estimates. Finally, for interaction models, we checked for animal-set-specific effects of strain rate by testing whether the simple slope for each animal set was different than zero using the simple_slopes function in reghelper (https://CRAN.R-project.org/package=reghelper). Statistical analysis and graphing were carried out in R version 4.4.2 (https://www.r-project.org/) using the tidyverse (https://www.tidyverse.org/). Graphs were generated using ggplot2 (https://ggplot2.tidyverse.org/). All model coefficients, means, and predicted values are presented along with their standard error, unless otherwise stated.

## Supplementary Material



10.1242/biolopen.062621_sup1 Supplementary information
