# Supplementary material for: The effect of strain rate on adhesion in the purple sea urchin (Strongylocentrotus purpuratus)
Source: Biol Open. 2026 Aug 27;15(8):bio062621. doi: 10.1242/bio.062621 (PMC13580561; doi:10.1242/bio.062621)
Supplement: Supplementary file 1 [file biolopen-15-062621-s1.pdf]

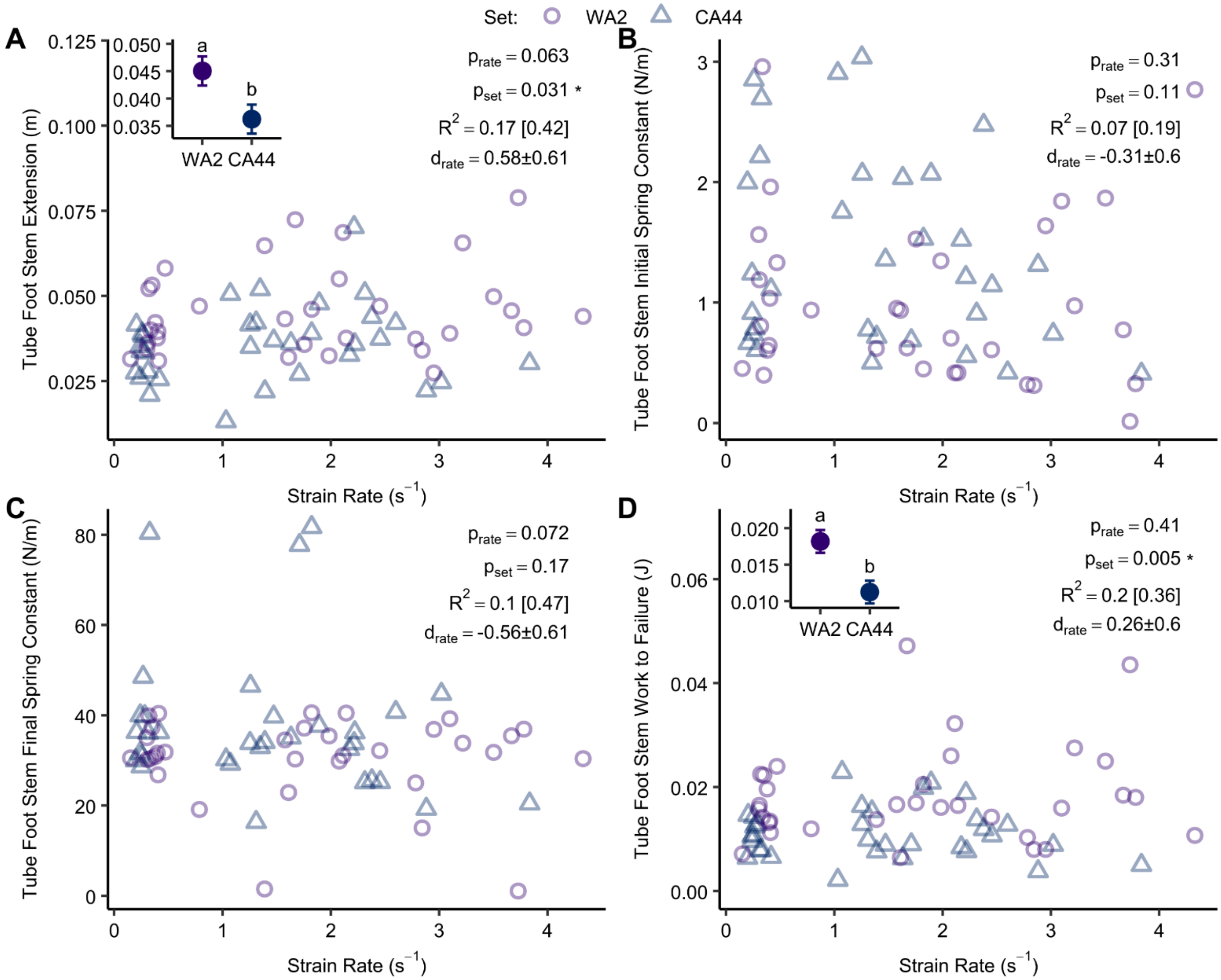


**Fig. S1. Strain rate has limited influence on tube foot stem material properties.** Extension (A), initial spring constant (B), final spring constant (C), and work to failure (D) are determined from a plot of tube foot stem length and force of extension (n=22). Extension is the length of the tube foot stem at breaking, initial and final spring constant are the slopes of the initial and final linear regions of the curve, and work to failure is the area under the length-force curve. Details for all variables can be found in text. When significant differences between animal sets are present for non-interaction models (A,C), insets show these differences (estimated marginal mean ± s.e.m.) at the mean strain rate (different letters denote statistically significant differences; post-hoc Tukey Tests, $p < 0.05$). Points are individual observations. Asterisks indicate statistically significant p-values ($p < 0.05$). Letters in the set names refer to collection location (CA: California, WA: Washington), numbers in the set names refer to the time spent out of their natural habitat prior to experiments in weeks. Full description of animal sets available in text. Complete statistical output is available in Table S1.

**Table S1. Linear mixed effects regression model parameters for relationships between tube foot stem material properties and strain rate.**

| Response | Form | n | Terms | F | df | p | $R^2_c[R^2_m]$ |
|---|---|---|---|---|---|---|---|
| Tube Foot Stem Extension (m) | rate + set | 22 | rate | 3.66 | 1,43 | 0.063 | 0.17[0.42] |
| | | | set | 5.41 | 1,20 | **0.031** | |
| Tube Foot Stem Initial Spring Constant (N/m) | rate + set | 22 | rate | 1.05 | 1,43 | 0.31 | 0.07[0.19] |
| | | | set | 2.81 | 1,20 | 0.11 | |
| Tube Foot Stem Final Spring Constant (N/m) | rate + set | 22 | rate | 3.4 | 1,43 | 0.072 | 0.1[0.47] |
| | | | set | 2.04 | 1,20 | 0.17 | |
| Tube Foot Stem Work to Failure (J) | rate + set | 22 | rate | 0.71 | 1,43 | 0.41 | 0.2[0.36] |
| | | | set | 9.92 | 1,20 | **0.005** | |

Both the marginal ($R^2_m$; fixed effects only) and conditional ($R^2_c$; full model) $R^2$ values are presented. n, number of individuals in the model.

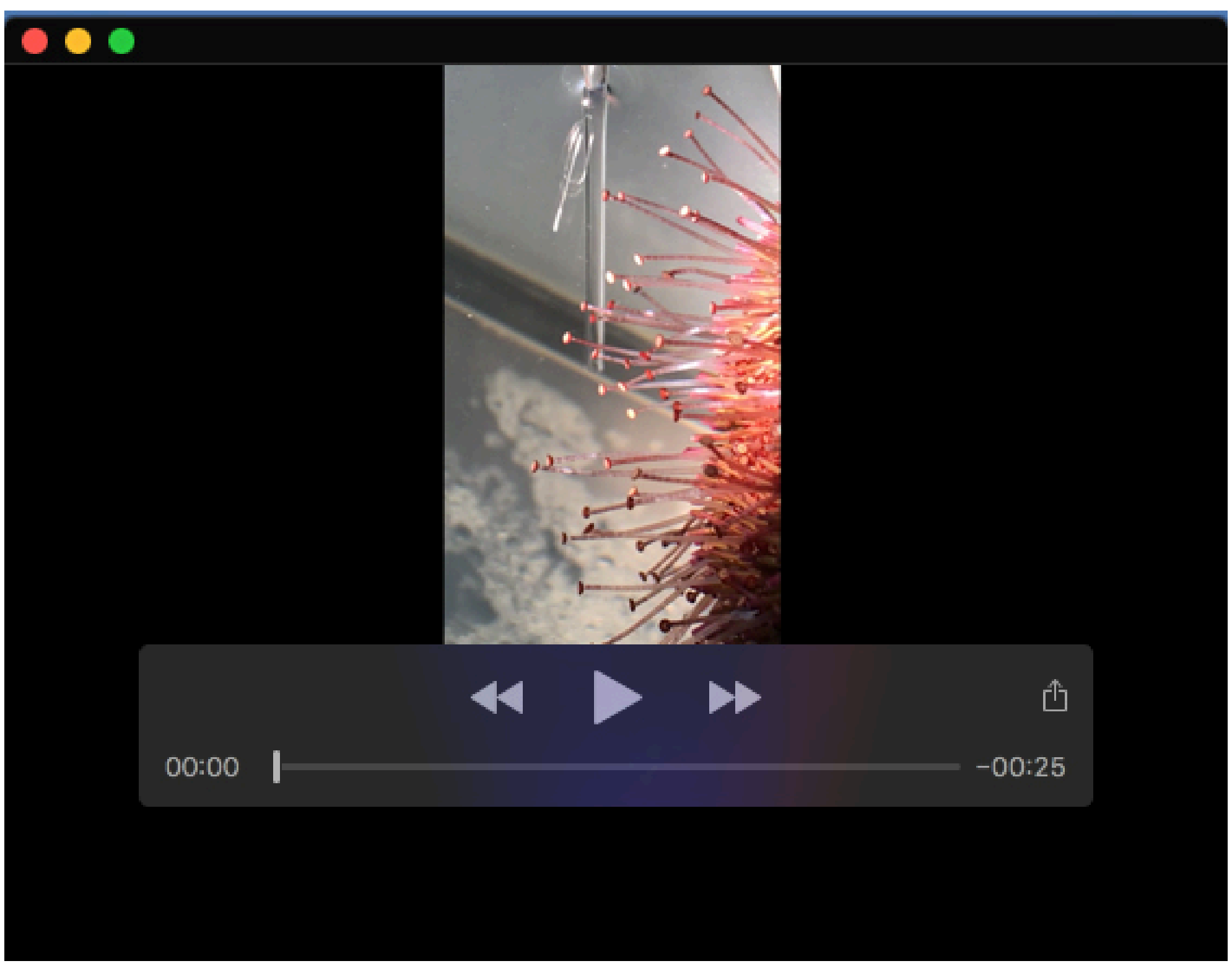


**Movie 1. Example sea urchin adhesion tests.** This video contains examples of single tube foot disc, tube foot stem, and whole animal adhesion tests.

Biology Open • Supplementary information
