# Supplementary material for: The effect of strain rate on adhesion in the purple sea urchin (Strongylocentrotus purpuratus)
Source: Biol Open. 2026 Aug 27;15(8):bio062621. doi: 10.1242/bio.062621 (PMC13580561; doi:10.1242/bio.062621)
Supplement: Supplementary file 2 [file biolopen-15-062621_review_history.pdf]

The effect of strain rate on adhesion in the purple sea urchin (Strongylocentrotus purpuratus)

Alyssa Y. Stark and Keegan Lutek
10.1242/bio.062621

Editor: Valentina di Santo

Review timeline

Original submission:	10 April 2026
Editorial decision:	18 April 2026
First revision received:	21 July 2026
Accepted:	23 July 2026

Original submission

First decision letter

MS ID#: bio.062621

MS Title: The effect of strain rate on adhesion in the purple sea urchin (Strongylocentrotus purpuratus)

Authors: Alyssa Y. Stark and Keegan Lutek

I have now reached a decision on the above manuscript.

The reviewer reports are shown at the bottom of this email.

As you will see, the reviewers gave favourable reports, but raised some critical points that will require amendments to your manuscript. I hope that you will be able to carry these out, because we would like to be able to accept your paper.

At this stage, we also ask you to ensure your manuscript complies with our formatting guidelines – please see our manuscript preparation guidelines for details. Provided you are able to fully address the referees' comments, we are positive about publication of your paper (we accept over 95% of revision submissions) and therefore hope you won't mind any extra work involved in reformatting your manuscript at this point.

Please upload both a 'clean' version of your Word file, along with a highlighted version clearly showing where you have made changes in the revised manuscript. Please avoid using 'Track changes' in Word files as these are lost in PDF conversion.

I should be grateful if you would also provide a point-by-point response detailing how you have dealt with the points raised by the reviewers in the 'Response to Reviewers' box. Please attend to all of the reviewers' comments. If you do not agree with any of their criticisms or suggestions please explain clearly why this is so.

Reviewer 1

Comments for the author

© 2026. Published by The Company of Biologists under the terms of the Creative Commons Attribution License (https://creativecommons.org/licenses/by/4.0/).

Peer review on Lutek & Stark submission to Biology Open:
The effect of strain rate on adhesion in the purple sea urchin (Strongylocentrotus purpuratus)

This manuscript describes experiments in which captured purple sea urchins were allowed to attach to glass substrates and the attachment number, size and mechanical performance under varying strain rates were measured. Although the tissues and the predominant matrix components such as collagen have strain-rate dependent behaviour, these experiments found limited, if any, strain-rate dependent mechanical behaviour in live, unanaesthetised urchins' tube feet.

The implications are two-fold: 1., that the behaviour of living organs and animals is not the same as the passive material components' behaviour and 2., that being alive and not anaesthetised may explain some of the lack of viscoelastic behaviour in whole tube feet - perhaps there is a nociceptive reflex response for example.

In general this is a well-written manuscript that places itself in the context of marine organisms' adhesion behaviour, and which does not overstep the limitations of the experimental design or observations in its interpretations, faithfully reporting null results and weak relationships where they are found. The lack of relationship is interesting, because they are against expectation based on prior work on tube foot tissues and anaesthetised animals; it seems that the next step would be to repeat the experiments to measure the effect of anaesthesia on tube foot mechanical behaviour. It is unfortunate that the 3 sub-samples are both from different locations and have different collection to experiment lag; this makes it difficult to tell whether the intergroup differences noted relate to the animals' collection location or their time out of the sea and in captivity, and whether some adaptation to captive conditions (and/or the sea conditions in the collection locations) has altered their behaviour. The authors interpret differences among the groups as being due to time in captivity, however, this is only one explanation and there isn't evidence to rule out other explanations.

There are a number of deficiencies in presentation that would improve the manuscript's value.

1. A figure detailing the experimental setup and giving some example image data would help a great deal. If there is video of some example tests, it would be very informative to understand the animals' behaviour during the experiments.
2. Briefly describe the image analysis; just writing "in Fiji" is not sufficient to understand or replicate the experiment.
3. Regression plots are somewhat confusing, in that each sample group is plotted independently with its own regression line, but then summary statistics of p, R^2 and d are provided, using all the pooled data (with sample set as a fixed effect), and not per group. It would help to plot the pooled regression line as well, which relates to the final summary statistics. The elevations of the sample set regression lines are helpful to illustrate differences among the sample sets
4. Regression lines should not exceed the limits of the data, in x and y. They only describe the measured data as a reduced model and cannot be used to predict (extrapolate or interpolate). Plotting a regression line where the slope is close to 0 and/or the p is high is of marginal value; the presence of the line on the plot implies a relationship that is not in the data. Reconsider plotting regressions where relationships are non-explanatory or do not differ from 0. The authors are correct to point out that although some relationships are "significant", they are not strong, in fact weak to negligible in magnitude. In the context of this kind of model fitting, low p (high significance) gives you confidence that there is not no relationship between variables (slope != 0), but the low R^2 tells us that the relationship found is very weak and that other factors explain nearly all the observed variance. To the authors' credit, they interpret their results with this in mind.
5. Data availability: R scripts, per-trial measurements and summary statistics are provided, however, the raw data are the images and mechanical test readouts (usually a series of force-displacement measurements used to construct a curve). Biology Open "encourages authors to make all data underlying their research available to the community" on a public repository, which the authors should do.

Biology Open reviewing rubric:

© 2026. Published by The Company of Biologists under the terms of the Creative Commons Attribution License (https://creativecommons.org/licenses/by/4.0/).

1. Experimental quality
a. Does each figure have the proper controls?
Yes
b. Are experiments performed using appropriate methods that will answer the question (or test the hypothesis or support the observations) posed by the authors? Is the right tool used for the job?
Yes
c. Were the data analysed using appropriate statistical tests?
Yes
2. Reproducibility
a. Were experiments in each figure performed using adequate number of biological replicates?
Yes
b. Is there sufficient raw data to assess the rigor of the analysis?
No - need raw data including images and force-displacement traces
c. Does the methods section provide sufficient detail to permit reproducibility?
No - need details on image analysis.
3. Completeness
a. Are the author's conclusions supported by the data?
Yes
b. Are there any flaws in the experimental design that invalidate the approach taken by the authors?
Yes - confounded source site and time in captivity
c. Are there experiments that have not been performed, but if true would disprove the conclusion? If yes, and if such experiments would be costly or time-consuming to perform, do the authors acknowledge this in a discussion of the limitations?
IT would be helpful to test directly the effect of anaesthesia on tube foot viscoelastic properties, but this could be a follow-on experiment.
4. Scholarship
a. Do the authors cite and discuss the merits of relevant data that would argue against their conclusion?
Yes
b. Do the authors cite and discuss the merits of relevant data that would support their conclusion?
Yes
c. For techniques/methods manuscripts, Do the authors cite and discuss the current state of the field and clearly explain how the method improves the field?
N/A

Reviewer 2

Comments for the author

The effect of strain rate on adhesion in the purple sea urchin (Strongylocentrotus purpuratus)

This study tests the strain rate dependence of tube foot mechanical properties in a strain rate regime higher than the strain rates imposed by ordinary podial movements but perhaps more appropriate to the higher strain rates an urchin might experience from wave forces. The study finds little strain dependence in this higher strain rate regime, whereas previous workers (e.g. Santos & Flammang, 2005, and Leddy and Johnson, 2000) found greater strain rate dependence, especially at lower strain rates and in anaesthetized animals (mutable collagen possibly softened). This result is very interesting for interpreting the ability of urchins to live in wave-swept environments. It also makes engineering sense in that the viscoelastic mutable collagen would thus function with little resistive force during daily slow extension activities of sea urchins using the collagen in its softened state. But the tube feet could generate higher forces and stiffness at the higher rates of loading from potentially dislodging wave forces. These higher resistive forces would be due to either behavioral modification of the mutable collagen or to a plateau of higher stiffness outside of the viscoelastic stiffening regime.
One topic that might warrant greater discussion is the possible role of helically oriented vs longitudinal collagen in the podial wall. The mutable collagen is probably in the longitudinally oriented layer whereas the helically wound layer might not be mutable collagen. As the tube foot extends, the helix angle becomes smaller and the fibers more aligned with the long axis of the tube foot. Thus, at larger extensions the helical fibers would take a larger fraction of the load relative

© 2026. Published by The Company of Biologists under the terms of the Creative Commons Attribution License (https://creativecommons.org/licenses/by/4.0/).

to the more strain rate dependent mutable collagen. During everyday functioning, during hydraulic inflation, the helical layer would range from 90 to 54 degrees, but during externally forced pulls, the angle could become less than 54 degrees and might contribute more to longitudinal resistance (because more closely aligned to longitudinal forces). The resistance of these helical fibers might be much less strain rate dependent because it is probably not mutable.
Overall this is an excellent, carefully done study, that builds nicely on extensive previous work on sea urchin tube feet and that has obvious importance for the function, ecology and evolution of sea urchins.
Detailed comments on the text.
* Line 75. Referring to load bearing, "Muscles, while present, likely contribute little, even when active." This is cited to (Santos & Flammang, 2005) but it would be useful to give a more precise guide than "little". I believe the paper they cited made a calculation that the muscles might contribute about 1% to the breaking strength, and at most 50% under the unlikely assumption that the tube foot muscle was as robust as anterior byssus muscle in mussels.
* Line 161 and 165, are the +/- ranges given standard deviations or standard errors or ranges?
* Line 165. The CA44 sea urchins have nearly identical diameter and height but much lower mass. Are these numbers correct? Are they significantly different? Is there any obvious reason that they would be so different?
* Line 170. The WA urchins have a very similar mass to the CA8 urchins but not to the CA44 urchins. This might suggest that the CA44 urchins are an unusual subset morphologically.
* Line 184 What is FIJI? Spell out at first use.
* Line 241. I don't understand the description of the area calculation. I might expect the use of the projected oral surface area which for approximately round urchins would be approximated by the area of a circle of diameter equal to the urchins diameter. So I don't understand the reference to an oblate spheroid. Although urchins are approximately oblate spheroids, I don't see how it figures into the calculation of projected oral surface area.
* Line 354. "strain rates that begin to approach those imposed by wave action". What strain rates are imposed by wave action and how do those rates compare with the tested strain rates?
* Table 1. I am not sure what Max Force means here. I do not think it was defined in the methods. Is it Max force of pulling a whole animal off?
* In Table 2, what are btf and fp? These terms are not defined elsewhere. I can guess that they are broken tube feet and footprints from reading the results, but they should be explicitly defined, perhaps most usefully in the table legend.
* I ran the R code. It ran flawlessly but I did get one error that caused no obvious problems; nevertheless, the authors might check whether they get this error as well.
Error in `annotate()`: ! Problem while setting up geom aesthetics. ℹ Error occurred in the 3rd layer. Caused by error in `list_sizes()`: ! `x$label` must be a vector, not an expression vector.

Reviewer's Responses to Questions

Experimental quality
Does each figure have the proper controls?
If 'No', please indicate reasons in Comments for Author box below.
Reviewer #1:
- Yes

Reviewer #2:
- Yes

Were the data analyzed using appropriate statistical tests?
If 'No', please indicate reasons in Comments for Author box below.
Reviewer #1:
- Yes

Reviewer #2:
- Yes

© 2026. Published by The Company of Biologists under the terms of the Creative Commons Attribution License (https://creativecommons.org/licenses/by/4.0/).

Reproducibility
Were experiments performed using adequate number of biological replicates?
If 'No', please indicate reasons in Comments for Author box below.
Reviewer #1:
- Yes

Reviewer #2:
- Yes

Does the methods section provide sufficient detail to permit reproducibility?
If 'No', please indicate reasons in Comments for Author box below.
Reviewer #1:
- No

Reviewer #2:
- Yes

Completeness
Are the manuscript's conclusions supported by the data?
If 'No', please indicate reasons in Comments for Author box below.
Reviewer #1:
- Yes

Reviewer #2:
- Yes

Scholarship
Do the authors cite and discuss the merits of data that would argue for and against their conclusion?
If 'No', please indicate reasons in Comments for Author box below.
Reviewer #1:
- Yes

Reviewer #2:
- Yes

Does the manuscript title & abstract accurately reflect the contents of the manuscript, without hyperbole?
If 'No', please indicate reasons in Comments for Author box below.
Reviewer #1:
- Yes

Reviewer #2:
- Yes

First revision

Author response to reviewers' comments

Note: All line references are to the highlighted copy of the manuscript.

Reviewer 1: Peer review on Lutek & Stark submission to Biology Open:

© 2026. Published by The Company of Biologists under the terms of the Creative Commons Attribution License (https://creativecommons.org/licenses/by/4.0/).

The effect of strain rate on adhesion in the purple sea urchin (*Strongylocentrotus purpuratus*)

This manuscript describes experiments in which captured purple sea urchins were allowed to attach to glass substrates and the attachment number, size and mechanical performance under varying strain rates were measured. Although the tissues and the predominant matrix components such as collagen have strain-rate dependent behaviour, these experiments found limited, if any, strain-rate dependent mechanical behaviour in live, unanaesthetised urchins' tube feet.

The implications are two-fold: 1., that the behaviour of living organs and animals is not the same as the passive material components' behaviour and 2., that being alive and not anaesthetised may explain some of the lack of viscoelastic behaviour in whole tube feet - perhaps there is a nociceptive reflex response for example.

In general this is a well-written manuscript that places itself in the context of marine organisms' adhesion behaviour, and which does not overstep the limitations of the experimental design or observations in its interpretations, faithfully reporting null results and weak relationships where they are found. The lack of relationship is interesting, because they are against expectation based on prior work on tube foot tissues and anaesthetised animals; it seems that the next step would be to repeat the experiments to measure the effect of anaesthesia on tube foot mechanical behaviour. It is unfortunate that the 3 sub-samples are both from different locations and have different collection to experiment lag; this makes it difficult to tell whether the intergroup differences noted relate to the animals' collection location or their time out of the sea and in captivity, and whether some adaptation to captive conditions (and/or the sea conditions in the collection locations) has altered their behaviour. The authors interpret differences among the groups as being due to time in captivity, however, this is only one explanation and there isn't evidence to rule out other explanations.
We agree with the reviewer that one cannot determine a specific cause for intergroup differences under the experimental design we used. While this means we cannot ascribe these differences to time out of their natural conditions, the results are still consistent with this hypothesis (though we agree that more rigorous testing is necessary, as we note on lines 299-302). We have clarified this confound between the animal sets in the discussion (lines 298-299) and use cautious language (e.g. “consistent with the hypotheses that…”, line 295) to avoid overstating our results.

There are a number of deficiencies in presentation that would improve the manuscript's value.

1. A figure detailing the experimental setup and giving some example image data would help a great deal. If there is video of some example tests, it would be very informative to understand the animals' behaviour during the experiments.
We appreciate this suggestion and believe it will clarify the methodology used in the paper. We have added a new Figure 1 that illustrates the experimental setups and have included a video of sample tests in a supplementary video (Movie S1).

2. Briefly describe the image analysis; just writing "in Fiji" is not sufficient to understand or replicate the experiment.
Thank you for noting this missing detail. The mention of “in Fiji” was on lines 84-85 of the previous version of the manuscript. This line was intended to describe the software (Fiji – an image processing software) used to perform the measurements now detailed on lines 361-368. To avoid confusion, we have: 1) moved the mention of this image processing software to the first mention of these measurements (lines 356-357), 2) added the appropriate reference here, and 3) included further details of the methodology for the counting of tube feet on line 441.

3. Regression plots are somewhat confusing, in that each sample group is plotted independently with its own regression line, but then summary statistics of p, R^2 and d are provided, using all the pooled data (with sample set as a fixed effect), and not per group. It would help to plot the pooled regression line as well, which relates to the final summary statistics. The elevations of the sample set regression lines are helpful to illustrate differences among the sample sets
Thank you for noting a lack of clarity in the presented statistics. We believe the reviewer is referring to the summary statistics presented in the former Figure 1 (which can also be found in Table 1). All the models in the former Figure 1 (now Figure 2) are non-interaction models. This means that the effect of strain rate (i.e. the slope of all lines and the effect size – d – for strain

© 2026. Published by The Company of Biologists under the terms of the Creative Commons Attribution License (https://creativecommons.org/licenses/by/4.0/).

rate) is the same for all animal sets and thus there is only one relevant p-value (and effect size) for strain rate. The p-value for animal set presented is from ANOVA output; there is only one relevant p-value here. Further, the R^2 value describes how the whole model fits the dataset, thus there is only ever one R^2 value expected for any given model (for mixed-effects models there are two ways to calculate this R^2 value [either including only fixed effects or including both fixed and random effects] as described in the methods, hence the two estimates presented). However, we agree that our former presentation can be clarified and we have done so as follows.

Panel A in the new Figure 2 illustrates no significant difference between the animal sets and so we have changed this panel to have a pooled regression line as suggested. Panels B-D illustrate a statistically significant effect of animal set, but not of strain rate. Thus, (keeping in line with comment 4 below) we have elected to remove the regression lines from these panels, but include insets that highlight these significant differences between the animal sets at the mean strain rate value. Note that because these are non-interaction models, the size of the difference between the sets remains the same at all values of strain rate. We have also highlighted all statistically significant p-values from the ANOVA output with asterisks to help clarify where differences exist. Similar changes have also been made to Figure S1 as the same kind of results are presented there.

4. Regression lines should not exceed the limits of the data, in x and y. They only describe the measured data as a reduced model and cannot be used to predict (extrapolate or interpolate). Plotting a regression line where the slope is close to 0 and/or the p is high is of marginal value; the presence of the line on the plot implies a relationship that is not in the data. Reconsider plotting regressions where relationships are non-explanatory or do not differ from 0. The authors are correct to point out that although some relationships are "significant", they are not strong, in fact weak to negligible in magnitude. In the context of this kind of model fitting, low p (high significance) gives you confidence that there is not no relationship between variables (slope != 0), but the low R^2 tells us that the relationship found is very weak and that other factors explain nearly all the observed variance. To the authors' credit, they interpret their results with this in mind.

Thank you for catching this mistake. This oversight (regression lines extending beyond the limits of the data for each animal set) has been corrected in the R code and in all figures.

We also appreciate the reviewer's suggestion not to include regression lines and have modified figures where there is not a significant effect of strain rate so that they do not include any regression lines. Differences between animal sets are now shown in an inset as described in the response to comment 3.

5. Data availability: R scripts, per-trial measurements and summary statistics are provided, however, the raw data are the images and mechanical test readouts (usually a series of force-displacement measurements used to construct a curve). Biology Open "encourages authors to make all data underlying their research available to the community" on a public repository, which the authors should do.

We have made the excel files which contain all force traces used in the present investigation available in a GitHub Repository (https://github.com/klutek/Lutek-and-Stark-2026-strainRate.git). This repository is currently set to Private, but will be made public at the time of publication. It can be accessed for review purposes at the following link: https://share-github.com/klutek/Lutek-and-Stark-2026-strainRate?access_token=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

Force traces were not recorded for the whole animal pull, so the data present in the per-trial measurements are the totality of data that was collected.

Biology Open reviewing rubric:

1. Experimental quality
a. Does each figure have the proper controls?
Yes
b. Are experiments performed using appropriate methods that will answer the question (or test the hypothesis or support the observations) posed by the authors? Is the right tool used for the job?
Yes
c. Were the data analysed using appropriate statistical tests?
Yes

© 2026. Published by The Company of Biologists under the terms of the Creative Commons Attribution License (https://creativecommons.org/licenses/by/4.0/).

2. Reproducibility
a. Were experiments in each figure performed using adequate number of biological replicates?
Yes
b. Is there sufficient raw data to assess the rigor of the analysis?
No - need raw data including images and force-displacement traces
These have been provided in a GitHub repository.
c. Does the methods section provide sufficient detail to permit reproducibility?
No - need details on image analysis.
This has been edited per the response to comment #2 above.
3. Completeness
a. Are the author's conclusions supported by the data?
Yes
b. Are there any flaws in the experimental design that invalidate the approach taken by the authors?
Yes - confounded source site and time in captivity
This has been addressed in the discussion per the general comments above.
c. Are there experiments that have not been performed, but if true would disprove the conclusion? If yes, and if such experiments would be costly or time-consuming to perform, do the authors acknowledge this in a discussion of the limitations?
IT would be helpful to test directly the effect of anaesthesia on tube foot viscoelastic properties, but this could be a follow-on experiment.
We agree that this would be helpful and we intend to perform this as a follow-up experiment, however, due to time and logistical constraints these were not possible at the time of data collection in the present manuscript.
4. Scholarship
a. Do the authors cite and discuss the merits of relevant data that would argue against their conclusion?
Yes
b. Do the authors cite and discuss the merits of relevant data that would support their conclusion?
Yes
c. For techniques/methods manuscripts, Do the authors cite and discuss the current state of the field and clearly explain how the method improves the field?
N/A

Reviewer 2: Review of Lutek and Stark,
The effect of strain rate on adhesion in the purple sea urchin (Strongylocentrotus purpuratus)

This study tests the strain rate dependence of tube foot mechanical properties in a strain rate regime higher than the strain rates imposed by ordinary podial movements but perhaps more appropriate to the higher strain rates an urchin might experience from wave forces. The study finds little strain dependence in this higher strain rate regime, whereas previous workers (e.g. Santos & Flammang, 2005, and Leddy and Johnson, 2000) found greater strain rate dependence, especially at lower strain rates and in anaesthetized animals (mutable collagen possibly softened). This result is very interesting for interpreting the ability of urchins to live in wave-swept environments. It also makes engineering sense in that the viscoelastic mutable collagen would thus function with little resistive force during daily slow extension activities of sea urchins using the collagen in its softened state. But the tube feet could generate higher forces and stiffness at the higher rates of loading from potentially dislodging wave forces. These higher resistive forces would be due to either behavioral modification of the mutable collagen or to a plateau of higher stiffness outside of the viscoelastic stiffening regime.
One topic that might warrant greater discussion is the possible role of helically oriented vs longitudinal collagen in the podial wall. The mutable collagen is probably in the longitudinally oriented layer whereas the helically wound layer might not be mutable collagen. As the tube foot extends, the helix angle becomes smaller and the fibers more aligned with the long axis of the tube foot. Thus, at larger extensions the helical fibers would take a larger fraction of the load relative to the more strain rate dependent mutable collagen. During everyday functioning, during hydraulic inflation, the helical layer would range from 90 to 54 degrees, but during externally forced pulls, the angle could become less than 54 degrees and might contribute more to longitudinal resistance (because more closely aligned to longitudinal forces). The resistance of these helical fibers might be much less strain rate dependent because it is probably not mutable.

© 2026. Published by The Company of Biologists under the terms of the Creative Commons Attribution License (https://creativecommons.org/licenses/by/4.0/).

We agree with the reviewer that the role of the two orientations of collagen fiber are of interest in this system. We have added mention and discussion of these fibers in the introduction (lines 93-95) and the discussion (lines 258-265). Note that if these collagen fibers behave as other polymers and biological materials generally do (as we suggest in the manuscript – lines 255-256), the helical (likely non-mutable) fibers are would be more strain rate dependent (not less strain rate dependent as the reviewer suggests) because they lack cross-links that could reduce the sliding of fibers past each other and are thus likely to display viscoelastic (i.e., rate dependent) properties. Unfortunately, we know of no direct test of whether mutable collagen behaves differently under different strain rates, so we have elected to keep our initial interpretation as there does not seem to be any direct evidence for either interpretation.

Overall this is an excellent, carefully done study, that builds nicely on extensive previous work on sea urchin tube feet and that has obvious importance for the function, ecology and evolution of sea urchins.

Detailed comments on the text.
* Line 75. Referring to load bearing, "Muscles, while present, likely contribute little, even when active." This is cited to (Santos & Flammang, 2005) but it would be useful to give a more precise guide than "little". I believe the paper they cited made a calculation that the muscles might contribute about 1% to the breaking strength, and at most 50% under the unlikely assumption that the tube foot muscle was as robust as anterior byssus muscle in mussels.
We agree that this additional context may be helpful and have edited the manuscript accordingly (lines 82-86 and line 267).

* Line 161 and 165, are the +/- ranges given standard deviations or standard errors or ranges?
These are standard error ranges. We have edited the text to make this clearer (lines 469-470).

* Line 165. The CA44 sea urchins have nearly identical diameter and height but much lower mass. Are these numbers correct? Are they significantly different? Is there any obvious reason that they would be so different?
We thank the reviewer for this astute observation. These values are indeed correct (and were measured using both the same procedure and by the same experimenter). While there is no statistically significant difference in mass between the CA44 urchins and either of the other animal sets (ANOVA, $F_{2,56} = 0.227$, $p = 0.80$) the apparent difference is likely due to this being the only batch of urchins that were shipped cross country prior to testing (e.g., use of nutritional reserves, stress).

* Line 170. The WA urchins have a very similar mass to the CA8 urchins but not to the CA44 urchins. This might suggest that the CA44 urchins are an unusual subset morphologically.
See comment for Line 165, we believe this is a result of transport and/or feeding. The details of these aspects of handling are found in the manuscript on lines 344-354.

* Line 184 What is FIJI? Spell out at first use.
FIJI is an open-source image processing software. This has been clarified and referenced accordingly in its first use on lines 356-357. The name FIJI is an acronym for "FIJI is Just ImageJ", but we (and perhaps others) find this to be a rather confusing full name and so have followed standard practice for this image analysis software and simply referenced the software, rather than indicating the full name.

* Line 241. I don't understand the description of the area calculation. I might expect the use of the projected oral surface area which for approximately round urchins would be approximated by the area of a circle of diameter equal to the urchins diameter. So I don't understand the reference to an oblate spheroid. Although urchins are approximately oblate spheroids, I don't see how it figures into the calculation of projected oral surface area.
This calculation is as first suggested by Stark et al., 2020. The intent is to capture the total possible area on the test of the sea urchin that could theoretically be used for adhesion to the substrate. We therefore first estimate the total surface area of the sea urchin test as an oblate spheroid and take half of this value (i.e. only the oral hemisphere of the test) as the total area that could theoretically be covered by the extensible oral tube feet used in adhesion.

© 2026. Published by The Company of Biologists under the terms of the Creative Commons Attribution License (https://creativecommons.org/licenses/by/4.0/).

* Line 354. "strain rates that begin to approach those imposed by wave action". What strain rates are imposed by wave action and how do those rates compare with the tested strain rates?
While there is no standard (range of) strain rate(s) for “those imposed by wave action”, Denny et al. (1998) characterize wave generated strain rates to be >> 1 s^{-1} (as listed on line 130). While our highest rates are not “much much greater than 1” (as is implied by Denny et al. 1998), they are indeed higher rates than previous investigations and so we characterize the rates used here as “approach[ing] those imposed by wave action”.

* Table 1. I am not sure what Max Force means here. I do not think it was defined in the methods. Is it Max force of pulling a whole animal off?
Thank you for catching this error, this has been changed both in text and in Table 1 to be “maximum adhesive force” for consistency. We have further double-checked our use of terminology for measured values to ensure they are clear and consistent throughout the manuscript. Changes to the main text, figures, and tables reflect this effort.

* In Table 2, what are btf and fp? These terms are not defined elsewhere. I can guess that they are broken tube feet and footprints from reading the results, but they should be explicitly defined, perhaps most usefully in the table legend.
Thank you for catching this oversight. We have added these definitions to the table legend for Table 2.

* I ran the R code. It ran flawlessly but I did get one error that caused no obvious problems; nevertheless, the authors might check whether they get this error as well.
Error in `annotate()`: ! Problem while setting up geom aesthetics. Error occurred in the 3rd layer. Caused by error in `list_sizes()`: ! `x$label` must be a vector, not an expression vector.
Thank you very much for taking the time to check the R code. We do not get this error when running the code, but as the reviewer notes, it does not cause any obvious problems because it only affects the presence or absence of annotations in the original Figure 1. Given the annotations appear normal we have not changed the code. If the reviewer can provide which line in the code is responsible for the error on their computer we are happy to try and find a universal solution.

Second decision letter

MS ID#: bio.062621R1

MS Title: The effect of strain rate on adhesion in the purple sea urchin (Strongylocentrotus purpuratus)

Authors: Alyssa Y. Stark and Keegan Lutek

I am happy to tell you that your manuscript has been accepted for publication in Biology Open, pending our standard publication integrity checks. It was accepted on 23rd July 2026.

© 2026. Published by The Company of Biologists under the terms of the Creative Commons Attribution License (https://creativecommons.org/licenses/by/4.0/).
